# Supplementary material for: Are carbon emissions trading and green financial instruments synergistic? -Comprehensive quantitative research based on content analysis
Source: PLoS One. 2024 Mar 7;19(3):e0298601. doi: 10.1371/journal.pone.0298601 (PMC10919601; doi:10.1371/journal.pone.0298601)
Supplement: S1 Data — (ZIP) [file pone.0298601.s001.zip › Data/地级市Z_F工作报告.zip!/╕ú╜¿/╚¬╓▌╩╨/└┤╫╘╣1⁄2╓┌║┼í╛┬φ┐╦╩2╛▌═°í┐_2012.docx]

各位代表：

　　现在，我代表泉州市人民政府向大会作工作报告，请予审议，并请政协委员和其他列席人员提出意见。

　　一、过去五年及2011年政府工作回顾

　　本届政府的五年，在中共泉州市委领导下，我市各级政府坚持以科学发展观为指导，扎实推进“两个加快”和跨越发展，完成十四届人大各次会议确定的目标任务，有效应对国际金融危机冲击，荣获改革开放、科技、品牌、卫生、环保、双拥、综治、普法、人防、文明创建等全国性荣誉称号，成为泉州经济社会快速发展、人民群众受益最多的时期之一。

　　——综合实力显著增强。五年来，我市生产总值、财政总收入、一般预算收入、规模以上工业产值、全社会固定资产投资年均分别增长14.1%、22.1%、20.6%、22.5%和26.8%，达4270.89亿元、500.36亿元、242.09亿元、8231.99亿元和1575.02亿元，均比2006年翻一番以上，经济总量连续13年位居全省首位；形成3个产值超千亿元、2个产值超500亿元产业集群；拥有产值亿元以上企业1497家、上市企业74家、中国驰名商标73件，分别是2006年的3倍、3.4倍和3倍。5个县（市）连续多年入围全国县域经济基本竞争力百强。

　　——城乡面貌显著改善。五年来，泉州城市发展从沿江向环湾推进，新一轮城市总体规划获批实施，城市建设全面提速，中心城区建成区面积161.5平方公里、城镇化率59.5%，分别比2006年增加81.5平方公里和11.5个百分点，海湾型城市框架初具雏形。累计抓好各级新农村试点示范村960个、扶贫开发重点村380个。组织实施水利工程、电网建设、水环境整治项目121个、186个和743个，新增公路、铁路里程3639公里和87公里，泉三高速、福厦高速（扩建）、福厦高铁（泉州段）等建成通车。

　　——改革开放显著加快。五年来，政府机构、规范行政自由裁量权、行政审批、简政放权等改革取得新成效；农村、国企、投融资、医药卫生等改革深入推进。全市累计完成外贸出口、实际利用外资分别达357.5亿美元、117.5亿美元，年均增长20.6%和13.4%。泉商“回归工程”实现投资370亿元。设立泉州台商投资区，新批2个国家级开发区和4个省级重点商品出口基地。泉州港和肖厝港成为首批两岸海上直航口岸；泉州晋江机场获准对外开放和成为对台直航点。

　　——人民生活显著提高。五年来，我市累计完成财政民生支出708.86亿元，年均增长24%。城镇居民人均可支配收入、农民人均纯收入分别达28703元和10578元，是2006年的1.8倍和1.6倍；实现城镇新增就业、农村劳动力转移就业78万人和32万人；基本医疗保障制度、城乡居民养老保险制度实现全覆盖；所有县（市、区）实现教育双高普九；孕产妇、婴儿死亡率分别下降74.8%和47.9%；海峡体育中心、泉州第一医院城东新院、梨园古典剧院等建成；闽南文化生态保护实验区设立，南音入选世界“非遗”名录；成功举办第六届全国农运会、第六届中国航海日、闽南文化节等活动；社会保持安定稳定。

　　2011年，全市按照市委“项目带动、转变提升、和谐进步、跨越发展”工作思路，全力以赴保增长、调结构、促跨越、重民生，实现了“十二五”开好局、起好步。

　　1、突出应对困难形势，力促经济平稳增长。针对经济下行压力加大，及时把保增长作为工作重点，采取一系列有效举措，帮助企业渡过难关。全市生产总值、财政总收入分别增长13.5%和25%。所有县（市、区）财政总收入均超10亿元。政企携手形成合力。市、县两级召开企业调研座谈会205场，帮助协调解决问题1900多个。坚持领导挂钩联系制度，加强对重点行业、重点企业和新增长点的服务扶持，支持民营龙头企业发挥支撑作用，亿元企业累计完成产值6771.41亿元，增长34.4%，占全部工业产值的74.6%；恒安、安踏、361°等企业税收贡献均超过5亿元。30位民营企业家联合发出倡议，留住总部机构。政策扶持全面展开。全力促进工业稳定增长，扶持小微企业发展，制订实施减轻企业负担的政策，减免行政事业性收费18项。出台自主创新、总部经济、动漫软件、工业设计、高新区建设等20多份政策文件。深入开展“送政策进企业”活动，帮助企业用好用足各项惠企政策措施。财政支持力度加大。全市财政安排上市、技改、创新、节能、引进人才、企业成长等扶持资金12.94亿元。帮助企业争取上级资金补助2.54亿元。生产要素保障有力。分行业、分领域开展政银企对接，每月筛选100家以上中小企业进行特别金融支持，中小企业贷款余额1820亿元；重视非银行金融机构的作用，累计为3700多家小微企业担保和发放贷款184亿元；新增上市企业14家，融资93亿元。获批农用地转用和土地征收5.97万亩，支持企业在规划许可前提下原址扩建、翻建厂房。兑现企业用工服务“17个确保”承诺。深入开展“送管理进企业”活动。完善共性研发、产品监测、科技信息等公共服务平台。市场开拓成效明显。成功举办泉州商品博览会系列重大经贸活动，石博会升格为省政府主办。鼓励企业在全国布局专卖店，拓展国内市场。组织企业抱团参加广交会、华交会、高交会等展会，新增出口超亿美元企业6家，外贸出口突破百亿美元大关，增长26%。项目支撑坚实有力。抓好455个工业重点技改项目，完成技改投资248亿元，增长23.4%。全面加强与央企的战略合作，积极参加省大型民企对接会、赴港澳重大经贸活动、“9·8”投洽会，取得显著成效，纳入省“三维”对接跟踪管理项目210个，总投资3719.25亿元。

　　2、深入实施“五大战役”，发展后劲持续增强。2154个“五大战役”项目完成投资1366亿元，完成年度计划123.8%。重点项目建设战役，418个重点项目完成投资670亿元。其中，南惠高速、南埔电厂二期等65个项目建成或部分建成；泉州湾跨海大桥、鸿山热电厂二期等120个项目开工，比年度计划增加50个。新增长区域发展战役，泉州湾、湄洲湾南岸、安永德三大增长区域完成全社会固定资产投资1393亿元。城市建设战役，236个项目完成投资270亿元。小城镇改革发展战役，21个试点小城镇947个项目完成投资330亿元。民生工程战役，553个项目完成投资96亿元。）“五大战役”的实施进一步夯实了我市跨越发展的基础。

　　3、加快转变发展方式，产业结构优化升级。科技人才作用更加凸显。召开企业创新千人大会，组织开展企业自主创新三年行动，落实财政科技经费5.82亿元，实施科技项目600多个，新增国家火炬计划重点高新技术企业8家、省级高新技术企业61家、创新型企业12家、省级以上研发机构10个、产业技术创新联盟5个、授权发明专利242项，制修订各类标准62项；国家级高新区管委会正式揭牌；高新技术产业产值1398亿元。颁布实施《泉州市中长期人才发展规划纲要》，成立引进高层次人才服务中心，启动6项重大人才政策和12项重点人才工程，新增5个院士专家工作站。先进制造业持续壮大。纺织鞋服、石油化工、装备制造、光电信息等行业产值增速均超过20%，泉州船厂修船、福橡合成橡胶等建成投产，泉州软件园、中国国际信息技术产业园、金保利、阳光大地、华锐风电等建设加快。品牌战略深入实施，新增中国驰名商标20件，我市品牌企业赞助世界大学生运动会各国代表团32个。现代服务业加快培育。港口货物、集装箱吞吐量分别达9330.48万吨和156.86万标箱，晋江陆地港、石湖石材物流园区投入运营，新增3A级以上物流企业9家。源和1916、锦绣庄、领show天地等文化创意产业园建成开园。实现社会消费品零售总额1424.76亿元，增长18.4%。完成旅游总收入288.4亿元，增长14.2%。泉台合作持续拓展。新批台资项目30个，实际利用台资2.08亿美元。台商投资区基础设施建设全面铺开。惠安国家级台湾农民创业园获批。成功举办第三届海峡论坛泉州系列活动和第四届海峡两岸农产品采购订货会。泉金客运航线运送旅客9.38万人次，泉州晋江机场实现对台春节包机直航，泉州港与高雄港结成合作港，泉台集装箱班轮货运直航实现常态化。泉台文化、教育等交流合作取得实质性进展。

　　4、强化水利设施建设，“三农”基础更加巩固。认真落实各项强农惠农政策，实施促进农民增收“六大工程”，完成农林水支出30.15亿元，增长42.6%，兑现补贴资金8.59亿元，农民人均纯收入增长13.8%。农业生产实现增产增效。粮食生产保持稳定。新增国家级农业产业化重点龙头企业2家、示范基地1个、农业“三品”认证30个和农民专业合作社390家。农林牧渔业产值267.22亿元。农村生产生活条件持续改善。实施水利项目58个，完成投资16.3亿元，白濑水利枢纽工程、晋江防洪工程前期工作加快推进，中小型水库除险加固、中小河流治理等工作完成年度任务，27.5万农村人口饮水安全问题得到解决，成为全国水资源综合管理改革试点城市。防汛抗旱成效明显。推进城乡环境卫生整治和家园清洁行动，完成农村改厕11.36万户，新增沼气用户2000户。实施“造福工程”搬迁7000人。植树造林34.32万亩，治理水土流失18.93万亩。新农村建设扎实推进。11个省派驻点村落实资金2000多万元，抓好市级新农村建设试点示范村20个和扶贫开发、老区重点村20个，示范效应持续扩大。小城镇改革发展不断深化。落实市级财政补助资金4500万元，新增3个省级、6个市级试点小城镇，完成所有试点镇总体规划修编，一大批现代产业、基础设施项目加快实施，试点镇示范带动作用更加明显。

　　5、全力推进环湾发展，城市建设步伐加快。开展“城市建设管理年”活动，317个项目完成投资358.03亿元。城市建设改造全面铺开。完成中心城区市政综合管廊、绿地系统等十大规划。东海、城东、北峰等新区建设加快推进，市行政中心完成搬迁；刺桐路改造、坪山路高架桥、洛江滨江大道竣工，东海隧道、后渚至城东通道将在春节前主线通车；东海经济适用房、晋江滨江商务区安置房等建设加快。浦西、龙头山、法石等39个“三旧”改造项目完成投资70.5亿元。进一步提升城市绿化美化水平，新增绿地342.55公顷，东湖、西湖公园水景喷泉竣工。城市环境整治深入实施。完成中心市区道路沥青化改造6条、电力线路缆化改造27条，新购公交车100辆，新增公厕121座。开展中心市区市容市貌专项整治行动，实施市容市貌项目23个，市区环境得到改善。城市管理水平不断提升。启动城市卫生管理考评工作，加大市政设施维护力度，加强城市管理行政执法，进一步规范物业管理，社区建设水平得到提高。

　　6、加强节能减排工作，生态建设成效明显。认真落实节能减排责任，全力做好国家环保模范城市复核迎检准备。节能降耗工作扎实推进。组织实施9个国家级重大节能项目和42个省级节能、循环经济项目，入选国家可再生能源建筑应用示范城市。环境污染整治力度加大。实施重点流域和近海水域环境综合整治项目141个，投入资金13.8亿元，近海水域功能区水质达标率77.8%；完成北渠水源综合整治工程，以及崇武至秀涂海岸带资源环境保护年度整治任务，抓好20条小流域污染治理，实施中心市区百源川池和内沟河清淤疏浚截污；“两江”上游水资源保护补偿专项资金提高到每年1亿元，12个集中式饮用水水源地Ⅲ类水质达标率保持100%。加强对重点行业污染整治，查处环境违法企业488家。环保基础设施不断完善。建成污水处理厂2座、管网178公里、垃圾转运站9座，城市污水、垃圾日处理能力分别达69.75万吨和5975吨。

　　7、着力保障改善民生，社会保持和谐稳定。就业和社会保障工作得到加强。全市新增城镇就业15.5万人，新增农村劳动力转移就业6万人，培训劳动者25.5万人次，城镇居民人均可支配收入增长14.1%。参加职工“五险”人数增加38.9万人次，失业、工伤和生育保险市级统筹稳步实施；参加新农合、城镇居民医保人数分别达560万人和165万人，人均财政补助均增至200元以上；城乡居民养老保险、社保卡制发应用走在全省前列；落实社会救助保障标准与物价上涨挂钩联动机制；建立社区居家养老服务中心100个、养老服务机构68家。全市慈善组织累计筹集善款21.6亿元，投入慈善救助活动14.9亿元；隆重表彰一批捐赠公益事业的华侨和慈善家。开工建设保障性安居工程45169套，超额完成省下达任务，保障低收入家庭住房困难户1.4万户，发放廉租住房租赁补贴1993万元；完成住房公积金归集29.7亿元、发放21.5亿元。出台落实稳定食品价格、保障市场供应措施，新增城市副食品调控基地99个，新设平价商店104个、直供销售点167个。社会事业稳步发展。建设31所公办幼儿园；中小学校舍安全工程开工面积51.56万平方米，新增义务教育标准化学校282所，中心市区增加优质义务教育学位1万个；保障外来务工人员随迁子女26万人平等接受义务教育；新增国家级重点职校2所，新组建职教集团3个；华侨大学新增一级学科博士点3个，泉州师院获批硕士点，泉州医高专被确定为国家示范性建设骨干高职院校，泉州幼师获批组建高等专科学校，我市14所高校与200多家亿元企业建立紧密合作关系。6个县（市、区）入选“中国民间文化艺术之乡”，新入选国家级非物质文化遗产名录3项，《董生与李氏》、《节妇吟》拍摄成数字电影，高甲戏剧团国家一级演员获梅花奖，《泉州文库》首批图书出版。成功承办世界女排大奖赛、第十届亚洲空手道锦标赛，举办国际南少林武术邀请赛、第九届市运会。获得全国文明荣誉称号25个。加快医药卫生体制改革，推进公共卫生服务均等化，基层医疗机构全面实施药品零差率销售，启动县级公立医院改革试点，新增床位数1726张。全市人口出生率11.66％。，政策符合率94.27%，生育关怀行动深入开展，推行流动人口基本公共服务均等化。持续做好第六次全国人口普查工作。社会管理创新扎实推进。深化新一轮平安泉州建设，开展“保平安创满意促和谐”专项行动，“清网行动”综合绩效考核位居全省第一；及时排查调处信访突出问题和矛盾纠纷；扎实推进安全生产标准化建设三年行动，四项指数全面下降；食品药品安全专项整治行动取得成效。有效防范民间借贷风险。政府其他工作全面推进。广播电视、民族宗教、信息化建设、国防动员、民兵、预备役、海防、人防、支前、双拥、统计、审计、口岸、方志、档案、气象、防震减灾和离退休干部服务、老龄、残疾人等工作取得新成绩。

　　8、促进机关效能提速，政府建设得到加强。自觉接受市人大及其常委会监督，认真执行市十三届人大七次会议和十四届人大五次会议议案决议，依法保障人大代表执行职务。支持市政协履行职能，主动接受政协委员民主监督。办理人大代表建议304件、政协委员提案465件，满意率均达100%。圆满完成11件34项为民办实事项目。实施“六五”普法规划。组织开展绩效评估暨“五大战役”考评工作，有效治庸治懒治散；启动新一轮深化简政放权工作，下放市级行政审批事权79项；建成行政电子综合监察系统，在全省率先推行网上远程协同评标。惩治和预防腐败体系不断完善。

　　回顾五年来的工作实践，我们深切体会到：必须坚持党的领导、服务大局，自觉在市委领导下开展政府工作，全力推进产业、港口、城市联动发展，积极在海西建设中凸显作为；必须坚持改革创新、彰显特色，立足泉州实际，敢于先行先试，发挥民营优势，突出政企互动，有为有效应对各种严峻挑战；必须坚持科学发展、跨越发展，致力在转方式上下功夫，在调结构上动真格，在转变中实现跨越，在跨越中促进转变；必须坚持民生优先、和谐进步，做到发展为了人民，发展依靠人民，发展成果惠及全市人民；必须坚持真抓实干、持之以恒，咬定发展不放松，一张蓝图绘到底，保持工作的连续性、稳定性、有效性，确保决策部署落到实处。

　　各位代表，五年的成绩来之不易，这是省委、省政府和市委正确领导，市人大、市政协大力支持，市政府历任领导持续推动，全市人民齐心协力、团结奋斗的结果。在此，我代表市政府向全市广大干部群众，各民主党派、工商联、无党派人士、各人民团体和离退休老同志，驻泉省部属单位、驻泉部队、武警官兵，企业界人士、来泉创业务工人员及广大港澳台同胞、海外侨胞、海内外朋友致以崇高的敬意！对人大代表、政协委员和全市人民给予政府的信任和支持表示衷心的感谢！

　　同时，我市发展中也存在不少问题和困难，主要表现在：经济“大而不强”，高新技术产业、现代服务业规模偏小，自主创新能力不强，资源环境制约明显，节能减排任务繁重；受外部宏观环境影响，企业生产成本上升，重点项目融资难度加大。城乡发展不够协调，中心城市集聚辐射功能不强，交通秩序、卫生保洁等问题不少；农村基础设施比较薄弱，农民持续增收压力较大。社会管理有待创新，事关群众切身利益的教育、医疗、住房、物价上涨、食品安全、饮水质量等问题亟待更好地解决，流动人口服务、社会治安等方面存在薄弱环节，安全生产形势仍较严峻，和谐社会建设任重道远。一些工作人员服务理念、服务效率跟不上发展的需要，机关作风仍需改进。2011年度26项指标中，第三产业增加值、物价涨幅控制未完成预期目标。对此，我们要采取更加有力的措施破解难题，以强烈的事业心和高度的责任感做好各项工作。

　　二、新一届政府工作及2012年工作目标任务

　　今后五年，是我市全面建成小康社会的关键时期。我们要大力弘扬“福建精神”，认真贯彻落实海西规划，按照市第十一次党代会作出的在更高起点上推进现代化泉州建设的战略部署，以更大的决心、更足的干劲，苦干五年、大干五年，把泉州建设成为经济总量大、产业结构优、发展后劲足的经济强市；建设成为人民生活质量好、文明程度高、公共服务完善、城市宜居宜业的美好家园。

　　政府工作指导思想是：以邓小平理论和“三个代表”重要思想为指导，深入贯彻落实科学发展观，按照省第九次党代会和市第十一次党代会部署，围绕在创新转型中创造新优势，以实施“两个加快”、推动跨越发展为主线，更加突出转变提升、民营经济、环湾发展、文化引领、民生优先、生态建设，做大做强产业、港口、城市，打造加快转变经济发展方式的示范区、县域城乡统筹发展的先行区，建设更加优美、更加和谐、更加幸福的现代化泉州。在实现“十二五”规划目标的基础上，力争至2016年，全市生产总值、财政总收入等主要指标比2011年翻一番，综合实力接近东部先进地区水平，在海西建设中持续走前列、挑大梁。

　　——推进产业创新转型，创造跨越发展新优势。始终坚持发展壮大实体经济，突出创新引领、人才支撑、产业升级，加快发展方式向集约型转变。推进民营企业“二次创业”，弘扬“晋江经验”，深入实施“百千万亿倍增计划”，建设全国民营经济创新发展示范区。组织实施“产业升级‘四化’工程”，力争至2016年传统产业品牌企业产值、成长型产业产值、战略性新兴产业产值分别达9000亿元、7500亿元、1000亿元，第三产业比重达40%以上。坚持投资和消费并重，深化与央企、民企、外企对接，全方位拓展与台港澳侨交流合作。加快泉州湾中心港区、湄洲湾南岸港区开发，完善现代立体交通网络，助推泉州跨越发展。

　　——推进城乡创新转型，创造统筹发展新优势。强化中心城市的辐射带动作用，提升城乡规划建设管理水平，加快城乡结构向一体化转变。围绕建设环泉州湾980平方公里中心城区，完善同城化发展机制，不失时机推进行政区划调整，规划建设一批环湾战略性、枢纽性、保障性基础设施项目，建好南、北翼新城，完善城市功能，凸显城市特色，提升城市品位，实现中心城区建成区面积达250平方公里，打造现代化海湾型城市。在厦漳泉大都市区建设中凸显作为。巩固壮大县域经济，有序推进沿海产业向山区县域梯度转移，支持安溪、永春、德化建设“泉州中心城市后花园”。深化小城镇综合改革，加快新农村建设，完善以工促农、以城带乡机制，促进农业增产、农民增收、农村发展。

　　——推进生态创新转型，创造可持续发展新优势。围绕促进经济与生态双赢，加快发展模式向低碳化转变。以对历史、对人民高度负责的态度，全面推行节能减排，集约利用土地、水资源，加快发展绿色低碳经济，增强可持续发展能力；完善生态补偿机制，突出“江河湖海”生态整治，抓好崇武至秀涂、崇武至泉港海岸带保护利用，加强重要生态功能区保护修复；深化“四绿”工程，森林覆盖率保持58.7%以上，建成国家级生态市。

　　——推进文化创新转型，创造协调发展新优势。发挥全国首批历史文化名城的优势，推动文化与经济社会共荣发展，加快文化大市向文化强市转变。深化文化体制改革，基本建成覆盖城乡的公共文化服务体系；加快文化产业发展，推进文化与市场、科技、旅游、工业融合，把文化产业培育成为支柱产业，力争文化产业增加值占地区生产总值比重达6%以上；加强文明建设，提高市民素质，争创全国文明城市。

　　——推进社会创新转型，创造和谐发展新优势。更加注重人的发展，加快社会管理向服务型转变。积极扩大就业，保持城乡居民收入年均增长10%以上。加快推进基本公共服务均等化，促进城乡教育、医疗、卫生等均衡发展。加强社会保障工作，积极发展老龄事业。大力创新社会管理，力争在流动人口、“两新”组织等服务管理上创造新经验，在“大调解”体系、防控体系、基层基础等建设上取得新进展。

　　2012年是新一届政府的第一年。我市发展主要预期目标为：地区生产总值增长12%；财政总收入增长18%，其中一般预算收入增长18%；规模以上工业产值增长21%；工业增加值增长13.6%；第三产业增加值增长11%；全社会固定资产投资增长20%；外贸出口增长10%；实际利用外资（历史可比口径）增长9%；社会消费品零售总额增长16%；居民消费价格总水平涨幅控制在4%左右；城镇居民人均可支配收入增长11%；农民人均纯收入增长11%；城镇登记失业率控制在2%以内；人口自然增长率控制在7.5％。以内；完成省下达的节能减排任务。各级政府各部门要按照市委决策部署，坚持稳中求进、着眼于进，把稳增长、控物价、调结构、惠民生、抓改革、促和谐更好地结合起来，强化工作落实，确保完成上述目标任务。

　　1、更加注重转变提升，加快培育创新力强的现代产业

　　实体经济是泉州的根基和优势。我们要以推进民营企业“二次创业”为突破口，发挥先进制造业的强大支撑，增强现代服务业的拉动作用，进一步提升实体经济发展水平。切实增强科技创新活力。深入开展企业自主创新三年行动，推进亿元企业与高校开展产学研战略协作，实施科技项目600个，新建产业技术创新联盟6个。建好国家级高新区，新增高新技术企业30家、创新型企业20家。争创国家知识产权示范城市。大力实施标准化战略，建设各类国家级质检中心。认真落实人才激励政策，研究实施民营企业集聚人才举措，支持建设院士专家工作站、博士后工作站等载体，培养引进高端人才和紧缺人才，用好技能人才和实用人才，促进大中专毕业生就业。致力打造先进制造业基地。实施“传统产业高端化工程”，支持民营龙头企业通过改制上市、资本运作、兼并重组、品牌建设，打造百亿集团和领军企业；开展“五年千项重点技改行动”，实施市级以上重点技改项目200项，抢占四大优势产业的高增值环节和高技术领域。实施“成长型产业集群化工程”，坚持抓龙头、铸链条，抓好中化炼油、联合石化脱瓶颈、石狮PTA二期等龙头项目，力争海峡轨道客车、玖龙纸业等投产，加快配套项目落地建设，推动四大成长型产业上下游一体化发展。

实施“新兴产业规模化工程”，抓好安溪湖头光电产业园等项目，重点突破LED芯片、竹纤维、新型建筑材料等技术，提高四大战略性新兴产业的规模生产力。要加快工业化与信息化融合，深化国家创新基金微波通信产业集群试点工作，推广应用对讲机模转数技术，发展物联网和云计算，争创国家新型工业化产业示范基地。大力发展海洋经济，抓紧做好布局规划和项目生成。深化国企改革，做大做强国有资产。开展“第三产业发展年”活动。实施“服务业现代化工程”，完善第三产业管理体制和配套政策，组织实施服务业重点项目91个。围绕构建现代物流体系，抓好中航三叶海西物流园、江南商贸物流中心等建设，推动制造企业剥离物流业务，培育引进3A级以上物流企业5家，大力发展港口物流。围绕构建现代市场体系，扩大泉州商品博览会影响，支持建设海峡石化产品交易市场、石狮国际轻纺城、中国水暖城三期等专业市场。围绕构建现代商贸体系，积极扩大消费需求，发展新兴消费业态，做好全国家政服务体系建设试点城市工作，积极培育现代商圈。围绕壮大商务服务业，培育一批税收超5000万元、超亿元的商务楼宇。围绕争创全国著名的旅游目的地，支持清源山创建5A级旅游景区，抓好18个旅游重点项目，办好陶瓷文化生态旅游节，推进旅游与文化深度融合，把旅游业培育成为主导产业。引导房地产业健康发展。完善中小企业公共服务体系。加大小微企业帮扶力度，推动大企业与中小企业协作配套、共同发展，支持行业协会、商会建设。充分发挥银行业金融机构的融资主体作用，加快首都银行（中国）有限公司泉州分行落户；规范壮大小额贷款、担保等非银行金融机构；关注民间借贷，防范地方金融风险。规划建设沿海向山区产业梯度转移基地；建好泉州开发区官桥园区。完成电网建设投资29.5亿元。出台促进产业工人“本地化”和鼓励创业的政策措施，支持企业建造员工公寓，破解用工难题。

　　2、更加注重以人为本，加快构建亲和力强的幸福家园

　　着力保障和改善民生，努力使广大人民群众学有所教、劳有所得、病有所医、老有所养、住有所居。抓好就业这个民生之本。完善就业培训制度，提高被征地、征海农民的就业技能，培训各类劳动者25万人次，实现城镇新增就业15万人，农村劳动力转移就业6万人。加强社会保障工作。继续完善城乡居民养老、医疗保障制度，新增城镇职工“五险”人数30万人以上。完善养老服务体系，建好泉州老年公寓、江南老年颐乐园及150个社区居家养老服务中心，建立老年服务志愿者组织。全力保持物价稳定，抓好“米袋子”、“菜篮子”建设，新建一批副食品调控基地，加强市场调节和价格监管。健全职工工资正常增长机制和支付保障机制。完善城乡一体的医疗救助制度，保障城乡困难群众生产生活。大力发展慈善事业。实施妇女儿童发展纲要。加大残疾人扶助力度。完善住房保障体系，开工保障性安居工程2.8万套，推进危房和石结构房屋改造。着力建设教育强市。确保2个区（市）通过“教育强县”省级评估验收，实现5个区（市）义务教育基本均衡发展。建设70所公办幼儿园。进一步扩大优质教育资源，加快城东学园建设和东海学园、江南学园开工，新增义务教育标准化学校290所，推广义务教育“小片区”管理模式，完成校安工程重建任务。加强校车安全管理。完善中小学心理健康教育网络。建好一批技能型紧缺人才培养基地、实训基地。支持我市高等院校发展。重视老年教育、特殊教育，完善终身教育体系。提升群众健康水平。深化医药卫生体制改革，抓好中医联合医院、泉州第一医院城东新院二期、二院东海新院等建设，新增床位数1700张；加强医疗人才的培养和引进，实现社区每万人有3名全科医生、每个卫生院有4名全科医生；扶持62家边远困难乡镇卫生院发展；稳步推进基层医疗卫生机构综合改革和公立医院改革试点；重视精神卫生工作。强化重大传染病防控举措，提升突发公共卫生事件处置能力。稳定低生育水平，充分发挥计生协会作用。广泛开展全民健身活动。真心实意为群众办事。着力解决群众关心的饮水、出行、家政、养老、住房、教育、健康、物价、治安等问题，切实抓好市区供水管网改造及水质优化工程、取消公交空调费、农村老年体育活动中心、青少年社会教育活动中心、贫困残疾儿童康复救助工程、为335户农村低保家庭建设安居房、“关爱女孩”五项工程、改造农村公路危桥、实施300公里农村公路安全保障工程、整治道路安全隐患、农村社区综合维修服务体系、地质灾害危险点搬迁安置等29项为民办实事项目，让广大人民群众得到更多实惠。

　　3、更加注重基础夯实，加快形成保障力强的“三农”格局

　　突出强农惠农富农，全市安排财政支农资金19.08亿元。提升现代农业。坚持“抓龙头、建基地、打品牌、拓市场、深加工、扶协会”，大力发展精细农业、设施农业和高效生态农业，实施"131"强龙工程，培育市级以上农民专业合作示范社40家，不断提高农业生产组织化程度。强化“五新”推广，引进农作物新品种160个，新增农产品“三品”认证22个。壮大提升茶产业，办好安溪茶学院。促进现代渔业增效增收。严格耕地保护，做好稳粮储粮调粮工作。完善重大动植物疫病防控体系，加强农产品标准化及质量安全工作。推广“世纪之村”信息服务平台，健全农业社会化服务体系。强化基础建设。抓好60个水利项目，争取白濑水利枢纽工程列入国家大中型水库建设规划，大力推进桃源、八峰、双溪、彭村等水库及台商投资区、晋江、石狮引水工程建设，解决30万农村人口饮水安全问题，确保水利建设投资25亿元以上。加强防汛抗旱工作。深化集体林权制度改革，植树造林33万亩，加强重要湿地保护和森林防火，建设森林泉州。实施新一轮农村电网改造升级工程。提级改造农村公路100公里以上。突出试点先行。深化省市试点小城镇改革发展，按照“较强的产业依托、发达的交通联系、完善的城市功能、优美的生态环境、相对规模的人口聚集、较高的政府管理水准”要求，进一步提高试点小城镇宜居宜业水平。建好20个市级新农村建设示范村、20个扶贫开发重点村、5个旧村居改造试点村、4个老区跨越发展工程重点帮扶村，带动提升全市新农村建设水平；加大“老少边岛”扶持力度，壮大村财收入。推进村庄规划全覆盖，深化城乡环境整治，实施一批“点”、“线”、“面”试点项目，深入开展家园清洁行动。

　　4、更加注重打好“五大战役”，加快拓展支撑力强的发展平台

　　积极拓宽领域和深化内涵。把农林水利、“四绿”工程、城镇美化、造福工程、饮水安全、防灾减灾等纳入“五大战役”实施范畴。重点项目建设战役要实施项目441个，其中在建项目298个、计划投资648.9亿元。新增长区域发展战役计划完成固定资产投资1606亿元。城市建设战役要实施项目159个、计划投资156.7亿元。小城镇改革发展战役要实施项目790个、计划投资269.5亿元。民生工程战役要实施项目403个、计划投资76.2亿元。深化“三维”项目对接。全力以赴引进大型央企、知名民企、世界500强投资，力争更多大项目、好项目列入省里盘子；按照“六个一定要”的要求，加强运作服务，紧盯项目不放，促进项目尽快落地投产。深化“回归工程”，吸引在外泉籍企业项目回归、总部回迁。有效保障战役实施。完善领导挂钩、分级管理制度，全力保障要素供给。做好项目前期工作，争取上级政策、资金、项目布局支持。加强资金运作管理，支持发行企业债券，提高项目融资能力。推进土地整理，实施填海造地工程。推行标准化管理，做到质量、安全、投资、环保和廉政五同步。

　　5、更加注重港口先导，加快完善竞争力强的开放体系

　　拓展港航综合交通。突出港口龙头作用，加强港口岸线规划管理和航道、港后区建设；抓好肖厝5#和6#泊位、石湖5#和6#泊位、秀涂16#泊位、小岞5万吨级码头、秀涂“人工岛”等项目建设；争取开通更多国际航线。加快完善立体交通网络，确保晋石、金安高速公路通车，加快南石、泉州湾跨海大桥、莆永、沈海复线、安溪连接线等建设进度，争取厦沙高速开工；加快湄洲湾南岸铁路支线、福厦铁路惠安站等建设；完成泉州晋江机场改建。巩固扩大泉台合作成果。加快泉州台商投资区开发开放，全力争取大型台资项目落地，打造高端产业聚集区；积极承接台湾光电、装备、石化以及现代农业、服务业转移，抓好旺旺福建总厂、迪斯腾太阳能电池以及国家级台湾农民创业园、台湾农业技术新品种推广中心、海峡西岸农产品物流中心等建设。拓宽对台客运、货运和小额贸易三条通道。办好闽南文化节、郑成功文化节，拓展两地民间交流领域。抢占国内外市场。借助异地商会和品牌优势，持续拓展国内营销网络，打造一批万店品牌专卖企业。出台扶持电子商务发展政策，培育一批国家电子商务示范企业，规划建设电子商务聚集区，用好国际电子商务平台，抢占网上市场份额。加快转变外贸发展方式，发挥泉州出口加工区作用；支持四个省级出口基地建设公共服务平台，赴境外设立品牌展示、仓储物流中心，建好德化国家级外贸出口转型示范基地；大力发展服务贸易，提高自主品牌和高附加值产品出口比重；加大新兴市场开拓力度，用好人民币跨境贸易结算试点政策，扩大出口信用保险和保单融资覆盖面；落实泉港泉澳经贸合作框架协定；办好品牌泉州香江行、海峡时装周等活动。加强大通关建设，引导更多产品从泉州口岸进出。支持企业“走出去”，鼓励开展跨国经营、收购国际品牌。加强外事侨务工作。广泛凝聚侨心、汇聚侨智、引进侨资。拓展对外交流合作渠道，密切与国际友城往来。

　　6、更加注重实施环湾战略，加快建设带动力强的中心城市

　　围绕做大做强环泉州湾中心城市，深化“城市建设管理年”活动，组织实施项目298个，年度投资300亿元以上，加快环湾同城化发展。构建环湾交通网络。启动环湾一环快速路、环城内环和外环快速路等重要交通节点的改造提升，抓好泉州大桥拓改、武荣大桥、金屿大桥、国道324丰洛段和晋鲤段、普贤路拓改、高铁站东西大道西延伸段、东海大街道路改造、机场连接线及环湾各片区市政道路建设，确保田安大桥、站前大桥建成通车。大力发展环湾公交，加快建设泉州高铁站综合客运交通枢纽，提高市区公交服务质量。深化交通综合整治。推进环湾全面开发。东海片区要抓好新工人文化宫、东海大厦等建设。城东双阳片区要尽快实施千亩商贸综合开发项目，抓好洛江中心商城等建设。江南池店片区要建好江南新城，加快池店城市建设步伐。泉州开发区要加快扩区发展、提升清濛园区。北峰丰州片区要抓好滞洪区、南启动区等建设。台商投资区要启动百崎湖东片区、秀涂城市生活配套服务区建设。晋江滨江片区要加快泉商大厦、世贸中心等进度。石狮环湾区域要加快蚶江、祥芝片区规划建设。南安霞美片区要抓好光机电贸展中心等项目。惠安崇武山霞片区要有序推进崇武古城保护开发。同时，大力推进泉港中心城区和安溪、永春、德化县城建设。组织实施一批“三旧”改造项目，高质量建设各安置小区。提升环湾城市功能。积极培育都市型产业，加快建设东海等总部经济区，推动设立中小企业营运中心。加强泉州古城保护开发，拓展文化旅游功能。实施中心市区绿化景观提升改造工程，建设泉州植物园、晋江两岸景观带主题公园、观音山、桃花山和滨海公园，新增绿地120公顷，创建国家生态园林城市。加强地下空间综合利用。建好一批城市综合体和高档酒店。强化环湾城市精细化管理。深入实施市容环境提升行动，及时做好道路破损、井盖丢失、路灯损坏的修复工作，整治汽车站、高铁站、机场周边环境，有效制止“两违”。加强城市管理综合考评和行政执法，建设数字化城管平台。抓好社区建设，提高物业管理水平。

　　7、更加注重低碳发展，加快构筑承载力强的生态环境

　　切实提升节能实效。大力推进清洁生产，抓好60个重点节能、循环经济项目，推动建陶、石材业转型升级；建成金井、小岞风电场。严格执行节能评估审查制度，出台用水用电差异化政策，坚决淘汰落后产能。切实提升减排实效。确保通过国家环保模范城市复核。深化重点排污单位环境保护主体责任自查自律行动。加强制革、印染、化工、电镀、造纸等行业深度治理，加大热电行业脱硫脱硝和老旧机动车淘汰力度。抓好6座城市污水处理厂、16座乡镇污水处理厂及配套管网、16个垃圾中转站及收集转运系统建设。切实提升环境整治实效。加强崇武至秀涂海岸带资源环境保护，深化近海水域、重点流域等环境污染综合整治，完成中心市区内沟河截污，落实生态环境补偿机制。强化农村环境连片整治。完成治理水土流失18.3万亩、“青山挂白”28.67万平方米。

　　8、更加注重文化引领，加快迈向软实力强的文化强市

　　加快发展公益性文化事业。实施文化惠民服务工程，加快闽南非物质文化遗产中心博览苑、泉州木偶剧院、泉州图书馆以及各类群众文化阵地建设。深入实施闽南文化生态保护实验区“十百千基础工程”，支持创作文化艺术精品，推动“海丝”、“闽南红砖建筑”和“侨批档案”申遗，办好“闹元宵·闽南文化周”，修订历史文化名镇、名村保护规划，加快《泉州市志》第二轮修编。大力发展文化产业。加大政策扶持力度，加快泉州当代艺术馆、T淘园、晋江创新创意创业产业园、南安创意产业基地等建设，办好首届创意产业节，举办行业性工业设计大赛，打造海峡工业设计中心，引进培育一批文化大企业大集团。抓好晋江、南安、石狮体育产业基地，以及各种国家级运动训练基地建设，加快体育产业发展。深化文化体制改革。加快文化、广电、新闻出版等融合发展，建立多元化投融资体制。加强文化市场建设，扩大文化消费。培育高素质文化人才队伍，重视发现培养扎根基层的乡土文化能人和非物质文化遗产传承人。致力提高市民素质。对照全国文明城市测评体系，广泛开展群众性精神文明创建活动和社会志愿服务活动；加强未成年人思想道德和大学生思想政治教育。强化新兴传媒运用管理；创办《香港泉州报》，举办“美国·中国泉州电视周”等活动，提升泉州文化的知名度和影响力。

　　9、更加注重管理创新，加快打造凝聚力强的和谐社会

　　维护社会安定。落实社会治安综合治理责任制，完善防控体系，重拳整治社会治安突出问题，提高人民群众对社会治安的满意率。严格落实依法处理信访事项“路线图”，深入开展接访活动，畅通群众合理诉求渠道，健全“大调解”体系。认真实施“六五”普法。保障社会安全。深入开展安全生产标准化建设三年行动，强化重点领域隐患排查治理，严防重特大安全事故发生。健全产品质量和食品药品安全监管机制，坚决打击商业欺诈、制假售假行为。完善公共安全预防预警体系，提升应急处置能力。促进社会和谐。创新流动人口服务管理，加强对特殊人群的帮扶和教育。引导社会组织健康发展。大力构建和谐劳动关系。推广和谐征迁工作法。落实民族政策，依法管理宗教事务。提升国防动员和双拥工作水平。贯彻军民融合发展方针，加强国防动员、民兵、预备役和海防、人防、打私工作，认真落实拥军优属和优抚安置政策，确保实现全国双拥模范城“七连冠”。

　　三、加强自身建设，打造勤廉兼优、为民高效的政府

　　新一届政府肩负着时代的重任，承载着人民的重托。经过30多年的发展，泉州正处于爬坡过坎、转变提升的关键时期，面临着高基数上实现高增长、快调整的巨大压力，面临着社会转型的一系列新挑战。我们必须以更高的工作标准，推动发展，服务人民，全力开创政府工作新局面。

　　解放思想、开拓创新。解放思想的空间越大，推动发展的空间就越大。我们要紧跟时代脉搏，树立世界眼光，强化战略思维，始终坚持抓全局性、导向性的大事。立足实际、发挥优势，用改革创新的思路，做足民营经济、文化名城、侨台资源等文章，形成泉州独特的核心竞争力。敢于负责、敢于担当，坚决破除一切束缚科学发展的障碍，切实用科学先进的理念、先行先试的胆识、行之有效的办法破解难题、推动工作，争当改革的先行者、创新的实践者。

　　鼓足干劲、奋发有为。越是复杂形势，越是严峻考验，越要迎难而上、锐意进取，充满激情地履职尽责；鼓实劲、出实招，真抓实干地开展工作。要有顽强拼搏的闯劲，在顺境中抢抓机遇，不迟疑、不松懈，乘势跨越；在逆境中坚定信心，不气馁、不言退，排难前进。要雷厉风行、高效运作，对议定的事、看准的事，就立说立行、一抓到底。要自我加压、奋力争先，不满足于一般化，以坚韧不拔的意志、比学赶超的气势，在各个岗位上创造新业绩，展示新形象、新作为。

　　执政为民、服务至上。人民政府的权力是人民赋予的。政府工作要始终把人民利益放在第一位，从群众最关心的事情抓起，从群众最困难的事情帮起，从群众最期望的事情做起。深化行政审批制度改革，能简则简、能快则快、能优则优，更好地服务群众、服务企业。腾出更多时间、更多精力，多做打基础、利长远、惠民生的好事实事。深化绩效管理考评，治庸治懒治浮治散，进一步提升政府执行力和公信力。

　　依法行政、清正廉洁。自觉接受人大法律监督、工作监督和政协民主监督，提高人大代表建议和政协委员提案的办理质量，充分听取各民主党派、工商联、无党派人士以及各人民团体的意见和建议，广泛接受新闻舆论和人民群众监督。完善决策机制，加强政务公开、行政复议、行政调解，建设法治政府。坚持艰苦创业、勤俭办事，把有限的资金用在刀刃上。全体政府工作人员特别是领导干部要率先垂范，模范遵守廉政准则，认真落实党风廉政建设责任制，健全惩治和预防腐败体系，树立清正廉洁形象。

　　各位代表，我们已经迈上科学发展、跨越发展新的历史征程。让我们在中共泉州市委的领导下，紧紧依靠和团结全市人民，万众一心，开拓进取，以更加优异的成绩迎接党的十八大胜利召开！
